# Commercial Determinants Drive Political Determinants of Health in a Neoliberal Society: Comment on "Energy as a Social and Commercial Determinant of Health: A Qualitative Study of Australian Policy"

**DOI:** 10.34172/ijhpm.8711

**Published:** 2025-04-16

**Authors:** Linda A. Selvey

**Affiliations:** School of Public Health, Faculty of Medicine, The University of Queensland, Brisbane, QLD, Australia.

**Keywords:** Australian Health Policy, Australian Energy Policy, Commercial Determinants of Health, Political Determinants of Health, Climate Change

## Abstract

Energy policies have a major impact on the health and well-being of the population. However, Australia’s energy policies rarely consider health and well-being in their policies. In Australia and in many other countries, energy policies, while developed by governments, are heavily influenced by commercial entities within the fossil fuel industry. This means that Australia’s energy policy does not reflect what climate science tells us is necessary for a safe climate. Australia’s environmental laws are insufficient to protect both nature and the environment. Environment and climate advocates have been urging the Australian government to strengthen these laws while industry, particularly the mining industry have been pushing to weaken them. This clearly demonstrates the strong intersection between commercial and political determinants of health.

 The World Health Organization’s (WHO’s) Commission on Social Determinants of Health developed a conceptual framework for the Social Determinants of Health that includes the political determinants (governance, macroeconomic policies, social policies, and public policies) in the structural determinants of health inequities. In the framework, these political determinants as well as cultural and societal values interact with other structural determinants such as social class, gender, ethnicity, education, occupation, and income that then influence intermediary determinants such as individuals’ material circumstances.^1^

 This framework does not include what is referred to as the commercial determinants of health. Commercial entities are responsible for many products and services that enhance health and well-being, but increasingly large multi-national companies have driven significant harm to human and ecological health through their production of goods and commodities and also through their influence on public policy and global economic systems.^2^

 The influence of multinational companies on global and national energy and environmental policy is a powerful example of these harms. These harms are being experienced around the world, as the impacts of climate change escalate. At the same time, global carbon emissions continue to rise. The World Meteorological Organization’s State of the Global Climate 2023 report is sobering. The year 2023 was the hottest on record, with the global average surface temperature 1.45 °C hotter than pre-industrial temperatures.^3^ This trend has continued into 2024 with every month from June 2023 to May 2024 inclusive being the hottest months on record.^4^ The health and social impacts of this increase in global average temperatures are being experienced world-wide through a range of impacts including severe heatwaves, storms, floods, sea level rise, wildfires, and droughts.^3^

 The lack of sufficient global action to reduce carbon emissions is in large part due to the influence of the fossil fuel industry. Since the early days of efforts to avert the worst of climate change, the industry has influenced politicians directly through lobbying and political donations and indirectly through affecting public opinion.^5-7^

 In Australia, climate policy is a polarising issue. After the most recent Australian federal election (in 2022), the centre-left Australian Labor Party increased Australia’s emissions reduction targets from a 26%-28% reduction of 2005 levels to a 43% reduction of 2005 levels by 2030. The current leader of the opposition, a member of the centre-right Liberal National party coalition recently announced that if they win the next Federal election they will “ditch” the current emissions reduction target and that they will not announce an alternative emissions reduction target before the next election.^8^ Many Australians are experiencing a cost of living crisis, because of high inflation, particularly for energy and housing costs. The Australian opposition leader is gaining traction in his rebuff of renewable energy because of high energy costs, claiming that renewables will increase the cost of energy and are unreliable.^8^ This demonstrates the tenuous nature of climate policy in Australia, even as global temperatures are rising to potentially dangerous levels.

 While Australia’s per-capita domestic emissions are among the highest in the world, our domestic emissions are a small fraction of our overall emissions, largely because of our coal and gas exports. The Intergovernmental Panel on Climate Change synthesis report AR6 and the International Energy Association Net Zero Roadmap both state that the world cannot afford to have any new coal, oil or gas projects if we wish to limit global warming to 1.5 °C above pre-industrial levels.^9,10^ In its net zero roadmap, the International Energy Association demonstrates how the world does not need any new fossil fuel projects for energy if the ambitious roadmap is followed.^9^ In spite of these clear warnings, the Australian Government, which signed off on the Paris Agreement to limit global warming to 1.5 °C above pre-industrial levels, continues to approve new coal and gas projects. This includes providing financial support to opening up massive new gas basins in the Northern Territory. At present, there is no requirement within Australia’s Environmental Protection and Biodiversity Conservation Act to consider climate impacts when assessing new projects. This was tested in a recent case (Living Wonders) initiated by the Environment Council of Central Queensland. This case contested 19 new or expanded coal, oil and gas projects on the basis of their potential impact on global climate. The case was lost in the Australian Federal Court and the proponents are now taking the case to the High Court of Australia.^11^

 The paper by Baum et al^12^ published in this issue, reports research investigating the inclusion of health and well-being in Australian energy policies. The context of their analysis was that energy is both a positive and negative social and commercial determinant of health. The study found that many policies do not explicitly mention either health or environmental impacts such as climate change and air pollution. This is despite the fact that energy is important for human health.

 Since 2002, Australia’s electricity system has transformed from publicly owned jurisdiction-based services to a national energy market with a number of providers, many of which are privately owned. The national energy market potentially provides stable electricity supply to a large proportion of the Australian population, but the focus has changed from energy as a public good to energy provision for commercial gain. High energy prices create inequities in energy access, which has consequences for people’s health, but these are not the only consideration of the energy markets.^12^

 The paper includes a conceptual framework for energy as social and commercial determinants of health. This framework includes policies as the overarching drivers of the characteristics of energy supply and consequential impacts on health (Figure 3).^12^ As policies are developed by governments, these policies could be considered to be political determinants of health.^13^ This is an important distinction because conceptualising the determinants as political directs advocacy towards where decisions are made. With the increasing influence of corporations on policy, however, the political determinants of health are being driven by the commercial determinants in the interests of benefits to commercial entities rather than to the community. This is apparent in a range of public health policy areas including food systems, tobacco, and alcohol policy.^13^ Important as such policies are, however, the commercial drivers of climate policy could arguably be considered to be the most critical for humanity, given that climate change is an existential threat to humanity and many other species.^10^

 In response to the challenges of influencing the political process, climate activists globally have been targeting the finance industry and contractors of fossil fuel companies to deter them from supporting the fossil fuel industry. As a result of this work, a number of banks in Australia and elsewhere have ruled out funding new fossil fuel projects and some contractors have refused to work on particular projects. In addition, these activities are eroding the social license of the fossil fuel industry. While these activities have been successful in eliminating some funding sources and removing some contractors, they have not been successful in stopping many projects completely. Other activists are mounting legal challenges to specific projects or directly targeting governments to require them to do more to protect our climate. Legal challenges can only be successful, however, if there is underpinning policy and legislation in support of their argument. As the Federal court found when considering the Living Wonders case, there is no legal requirement of Australia’s environment minister to consider emissions when making decisions about fossil fuel projects.

 Influencing political policy in opposition to the demands of the fossil fuel industry is very difficult, particularly in a bipartisan political environment such as in Australia. Australia’s Environmental Protection and Biodiversity Conservation Act is currently under review and the Australian Labor party promised in an election commitment to strengthen the legislation in this term of government. The laws need to be strengthened to protect nature and the climate, including the introduction of a “climate trigger,” which would allow consideration of climate impacts in decisions about fossil fuel projects. The Australian government has reneged on this promise, ostensibly in response to lobbying by the fossil fuel industry.^14^ As a result a number of different environmental and climate organisations are mobilising their constituencies to target Labor members of parliament. Whether this will be strong enough to withstand the might of the Fossil fuel industry remains to be seen. Mobilising people around climate change during a cost of living crisis is a challenge, but what do we have to lose but a liveable world!

## Ethical issues

 Not applicable.

## Conflicts of interest

 The author volunteers with a number of climate organisations in Australia.
